# The color of fruits in photographs and still life paintings

**DOI:** 10.1167/jov.24.5.1

**Published:** 2024-05-01

**Authors:** Thorsten Hansen, Bevil R. Conway

**Affiliations:** 1 Department of Psychology, Justus Liebig University Giessen, Giessen, Germany; 2 Laboratory of Sensorimotor Research, National Institutes of Health, Bethesda, MD, USA

**Keywords:** painting, art, color, memory, still life

## Abstract

Still life paintings comprise a wealth of data on visual perception. Prior work has shown that the color statistics of objects show a marked bias for warm colors. Here, we ask about the relative chromatic contrast of these object-associated colors compared with background colors in still life paintings. We reasoned that, owing to the memory color effect, where the color of familiar objects is perceived more saturated, warm colors will be relatively more saturated than cool colors in still life paintings as compared with photographs. We analyzed color in 108 slides of still life paintings of fruit from the teaching slide collection of the Fogg University Art Museum and 41 color-calibrated photographs of fruit from the McGill data set. The results show that the relatively higher chromatic contrast of warm colors was greater for paintings compared with photographs, consistent with the hypothesis.

## Introduction

Color is both a stimulus and a response. As a stimulus, color starts with the transduction of visible light by three types of cone photoreceptors (Stockman & Brainard, 2010). These retinal signals are then processed by an extensive network that engages subcortical circuits and many regions of the cerebral cortex that interpret the retinal signals in the context of prior experience, expectations, and behavioral objectives (Conway, 2009; Conway, 2018). Conscious experience is presumably the outcome of this neural activation; and color provides a promising tool for exploring the linkage of neural activity and consciousness (Bannert & Bartels, 2018; Elliott & Cao, 2013; Siuda-Krzywicka & Bartolomeo, 2020; Witzel & Gegenfurtner, 2018). Herein we analyze the way color is represented in still life paintings to test how prior knowledge impacts color perception. We include an analysis of the variance of chromatic and luminance contrast (Penacchio, Haigh, Ross, Ferguson, & Wilkins, 2021).

Subjects who are asked to reproduce the color of familiar objects consistently choose a color that is more characteristic of the dominant chromatic attribute of the object (Bartleson, 1960). This “memory color” is typically more saturated than the color of real objects (Hunt, 1987; Newhall, Burnham, & Clark, 1957; Siple & Springer, 1983), and may modulate perception of objectively rendered images of familiar objects. For example, an achromatic photograph of a banana might appear to some observers as slightly yellowish (Hansen, Olkkonen, Walter, & Gegenfurtner, 2006). Such “memory color effects” were suggested by Hermann Helmholtz and later invoked by Ewald Hering, who described objects as appearing through the “spectacles of memory colors” (Yendrikhovskij, Blommaert, & de Ridder, 1999).

The memory color effect is evidence of what might be a more general computational goal of vision, that of idealization, by which the brain computes an idealized concept that reflects the distinct, diagnostic features of behaviorally relevant objects (Martin, 2007). Accordingly, the concept of “banana” is defined by both a crescent shape and the color yellow, and it may reside in the mind's eye as more crescent-like and more saturated than any real banana (Bartleson, 1960; Tanaka, Weiskopf, & Williams, 2001). When tasked with adjusting an image of a banana to appear gray, participants tend to render it objectively bluish (Hansen et al., 2006), as if some subtle color complementary to the normal color of the banana is required to counteract the memory color effect that tints the perception of an achromatic banana slightly yellowish. The veracity of memory color effects has been challenged (Firestone & Scholl, 2016), but these effects have been reproduced, albeit with variable expression depending on the realism of the rendering of shape and texture (Olkkonen, Hansen, & Gegenfurtner, 2008; Vurro, Ling, & Hurlbert, 2013) and the extent to which the colors of the objects align with the daylight locus (Witzel, Valkova, Hansen, & Gegenfurtner, 2011).

We sought to test the impact of color–shape knowledge on still life painting, reasoning that, during the act of painting, the artist must hold in memory, even if for brief periods of time, the colors associated with the fruits they are painting. We hypothesize that artists are painting these memories, and because the colors of memories are more vivid than those in real life, we predicted that the subject of the still life paintings—the fruit—will have relatively higher saturation than real fruit. This hypothesis can be understood by analogy with the over emphasis of lines in drawings (Conway, 2022). The visual system encodes object shapes by their boundaries, so the ideas of objects in our minds are defined by lines, which then explains why lines can be used to represent object shape (Livingstone, 2002; Sayim & Cavanagh, 2011). The consequence is that lines are more prominent in rendered depictions than they are in the retinal image. Here, we argue that an object concept is defined not only by its distinct form (represented by lines), but also by its specific hue (impressed in the mind by its saturation), and these two aspects of the concept leave a trace in the artists’ work. The parts of scenes labeled as objects are more likely to be warm colored compared with backgrounds (Ling, Vurro, & Hurlbert, 2008; Rosenthal et al., 2018). We leverage this observation to test the hypothesis, using warm-colors as a proxy of “fruit” and cool colors as a proxy for “backgrounds”. We find that the chromatic contrast (or chroma) of warm-colored pixels compared with other pixels is higher in paintings compared with photographs of fruit.

## Methods

### Image data sets

We used images from two data sets of fruits in still life paintings and photographs: The images of still life paintings were obtained from the Fogg Art Museum and the photographs of the fruits were obtained from the McGill calibrated color image database (Olmos & Kingdom, 2004). The Fogg Art Museum is attached to Harvard University; the slides are used in art history lectures and were generated by art historians to accurately represent the color in the paintings. We attempted to identify all slides in the collection that were of paintings depicting fruit, regardless of date, artist, style, or location. The slides were digitized for further analysis using a Nikon LS 4000 ED (Super Coolscan 4000 ED) film scanner and stored as TIFF images. We cropped the images to remove any frame or outside border and stored them as PNG images; all remaining pixels in the slides were included in the analysis. We used 108 images of paintings as detailed in Appendix A.

The McGill calibrated color image database contains 708 images that are 768 × 576 pixels, grouped into nine categories: animals, flowers, foliage, fruits, land and water, man-made, shadows, snow, and textures. We selected just the 41 images of fruit for analysis. The images are stored as standard RGB images in TIFF.

### Color space

Images of both data sets were analyzed in the same way. Some of these details have been described previously (Hansen & Gegenfurtner, 2009) and are paraphrased here for ease of reference. First, we converted RGB images to the DKL color space. DKL is a cone-opponent space of an achromatic signal L/M/S and two chromatic signals L/M and S, corresponding with the chromatic preferences of retinal ganglion cells and cells in the lateral geniculate nucleus (Derrington, Krauskopf, & Lennie, 1984). Next, we computed joint histograms for each of the three possible pairs of image planes L/M/S and L/M, L/M/S and S, and the isoluminant plane L/M and S. Note that there are two common naming conventions for the DKL axes, either in terms of lights or mechanisms. We used the lights convention here instead of the mechanisms convention where the axes are labeled L + M, L − M and S – (L + M).

### Joint histograms

We computed the joint histograms by discretizing the DKL plane into 128 × 128 bins ranging from −2 to 2 along each axis, projecting all image pixels represented in DKL to the respective plane and counting the number of pixels that fall into each bin. We normalized the joint histograms by the number of pixels in the image such that the histograms become independent of image size. For each data set, we computed joint histograms for each image and the mean histogram for all images for each of the three DKL planes. We also computed a “chromogram,” where we colored each bin with the mean of all pixels that fall into the bin (Figure 2, second row).

### Chromatic contrast curves

To further analyze and compare the histograms we computed chromatic contrast curves for different quadrants of the histograms. To compute chromatic contrast curves, we computed the chromatic contrast for each bin as the distance to the origin (0, 0) and then summed the bins with the same chromatic contrast. We rounded the chromatic contrast to the 4th significant digit, resulting in 1,464 unique values of the chromatic contrast. The chromatic contrast curve shows for each chromatic contrast in the plane the relative number of pixels in the image. We normalized the chromatic contrast curves with the number of bins in the given quadrant. This allows us to compare quadrants of different sizes, for example, the quadrant 1 against the combined quadrants 2, 3, and 4. Finally, contrast curves were smoothed by convolution with a 1-D Gaussian with a standard deviation of 10.

### Confidence intervals for chromatic contrast curves

We used a bootstrap analysis to compute confidence intervals for chromatic contrast curves (Figures 3D and E, 5D and E, and 6D and E). First, we computed the chromatic contrast curves for each image. Next, we randomly sampled *N* chromatic contrast curves (*N* being the number of images in the data set) and computed the mean chromatic contrast curve. This process was repeated 1,000 times. From the resulting 1,000 mean chromatic contrast curves we compute the 95% confidence interval by sorting the curves and picking the 25th and 975th curves.

### Segmentation of the fruits

The cut-out of the fruits shown in Figure 4 were generated manually with Matlab's Image Segmenter and method “Draw ROIs” (regions of interest).

### Analysis of local chromatic and achromatic differences

The analysis of the achromatic and chromatic contrast follows the procedure described by Penaccchio et al. (2021) to compute chromaticity differences. First, we cropped the central square of the image and resized it to 256 × 256 pixels using The Matlab Inc (2019) function imresize with bicubic interpolation. Next, we converted the images to the CIE LUV color space using Matlab's functions makecform (with arguments srgb2xyz and xyz2upvpl) and applycform. We computed the local chromatic difference as the mean Euclidean distance between the central pixel and its eight neighboring pixels in the (u′, v′) plane. Similarly, we computed the local achromatic difference as the mean Euclidean distance between the central pixel and its eight neighboring pixels in the L plane. Finally, we computed the average chromatic and achromatic difference for each image as the mean local difference of all pixels.

The images and analysis scripts are provided at doi:10.6084/m9.figshare.25504717.

## Results

**Figure 1.**
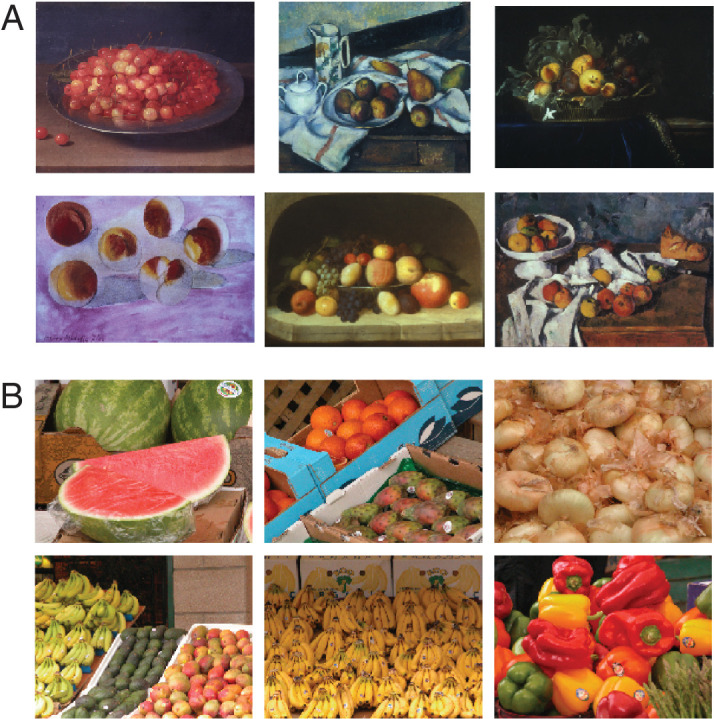
Sample images of the fruit paintings (A) and photographs (B).

We analyzed color in 108 slides of still life paintings (Table S1 provides the list of works), and 41 color-calibrated photographs of fruit from the McGill Color Calibrated Database (see Methods), examples of which are shown in Figure 1.

**Figure 2.**
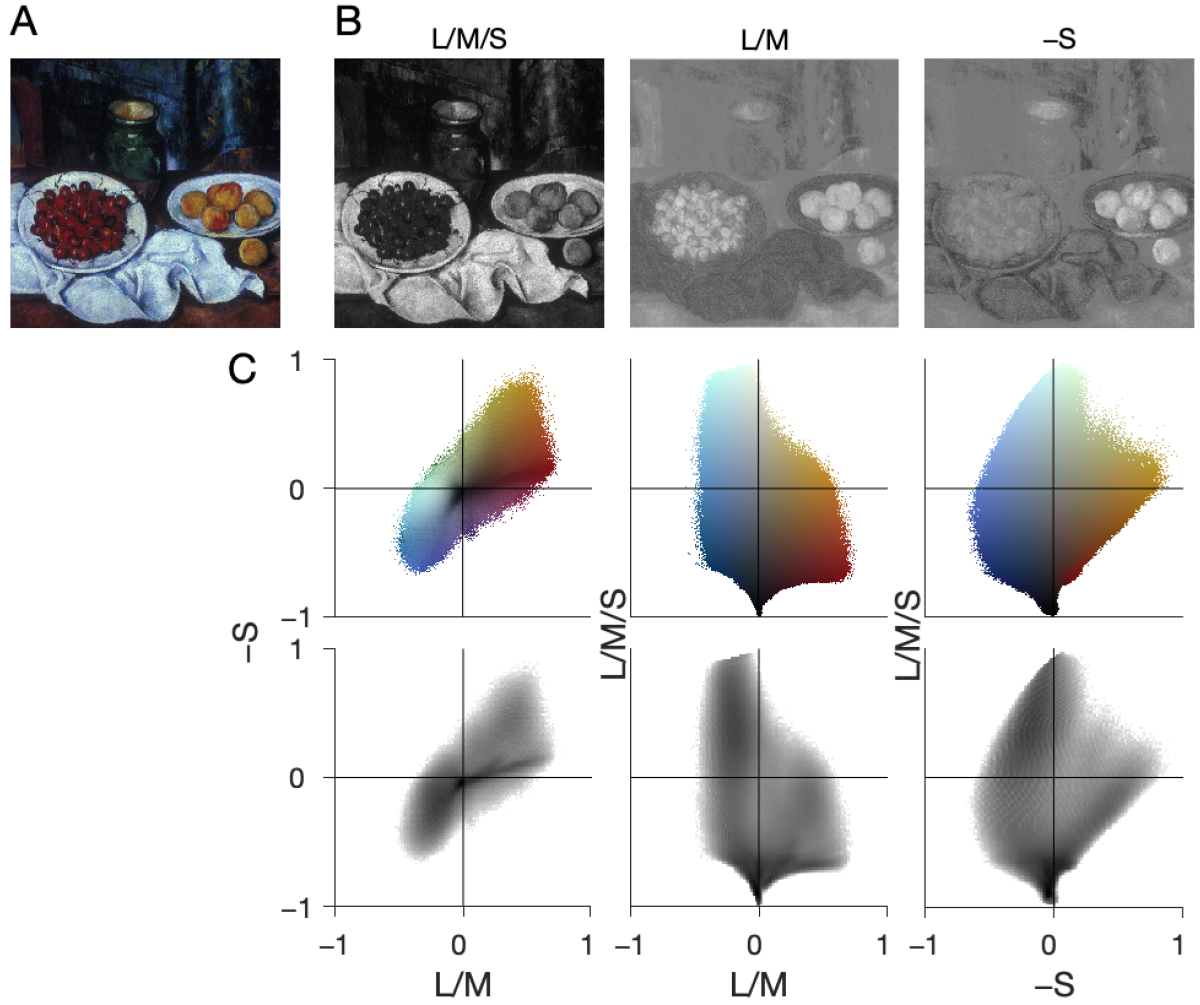
Images analysis for a sample image. (A) Input image. (B) Activation along the three axes of DKL space: achromatic L/M/S, chromatic teal-cherry L/M, chromatic violet-lime S. (C) Joint distribution of image pixels in the three possible pairs of DKL axes. (Top) The color is the mean of the colors of all pixels of the input image that fall into the respective bin. (Bottom) The gray level denotes the number of pixels of the input image that fall into the respective bin. The darker the gray level, the more pixels fall into the bin.

The pixel values of each image, photograph or painting, were analyzed by transforming the chromaticity values into the cone-opponent DKL color space that the retina and lateral geniculate nucleus are thought to use to encode the cone signals (see Methods for details). We used the DKL space because the axis are defined in a physiologically meaningful way and it is well-suited for predicting saturation in natural scenes (Schiller & Gegenfurtner, 2016). Figure 2 shows this analysis for a still life painting by Cezanne (*Still Life with Cherries and Peaches*, 1887). The top row shows the input image as represented by the two chromatic axes L/M and S and the achromatic axis L/M/S of the DKL color space. The second row shows the distribution of the mean colors in the image projected to the three cardinal planes of DKL. The painting did not have a uniform representation of colors across color space. Instead, there were more pixels with colors in quadrants 1 and 3 of plots with axes of S versus L/M (left), and more dark colors than light colors (middle). This distribution of colors is similar to biases found in natural images (Webster & Mollon, 1997).

**Figure 3.**
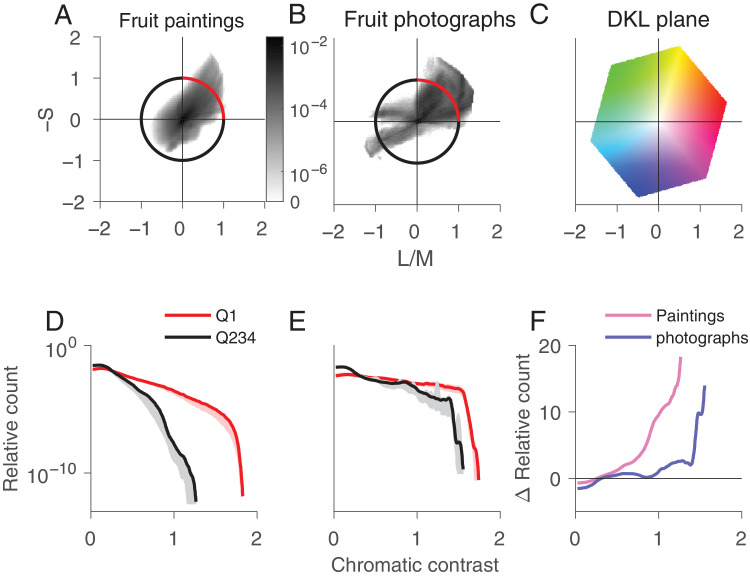
Analysis of color distribution in the DKL isoluminant plane for paintings vs. photographs of fruits. Joint histogram for all paintings (A) and the photographs (B). The unit circle is color-coded with the two sets of quadrants analyzed in (D) and (E). (C) Projection of the RGB color cube to the L/M–S plane. Normalized histogram of chromatic contrast for the "fruit" quadrant Q1 (red) vs. the other three quadrants Q234 (black) for the paintings (D) and the photographs (E). The light red and gray shaded areas indicate the 95% confidence interval. (F) Difference between the red and black curves in (D) and (E) for the paintings (pink) and photographs (blue).

To investigate the distribution of fruit color in paintings and photographs, we computed the joint histograms for the two chromatic DKL axes L/M and S, averaged across all paintings (Figure 3A) and photographs (Figure 3B). The joint histograms shown throughout the article bin every pixel of the image as projected to the respective plane. We averaged the individual joint histograms by taking the mean of each bin. In both paintings and photographs, there is a strong bias for pixels in quadrants 1 and 3, as for the example image (Figure 2).

**Figure 4.**
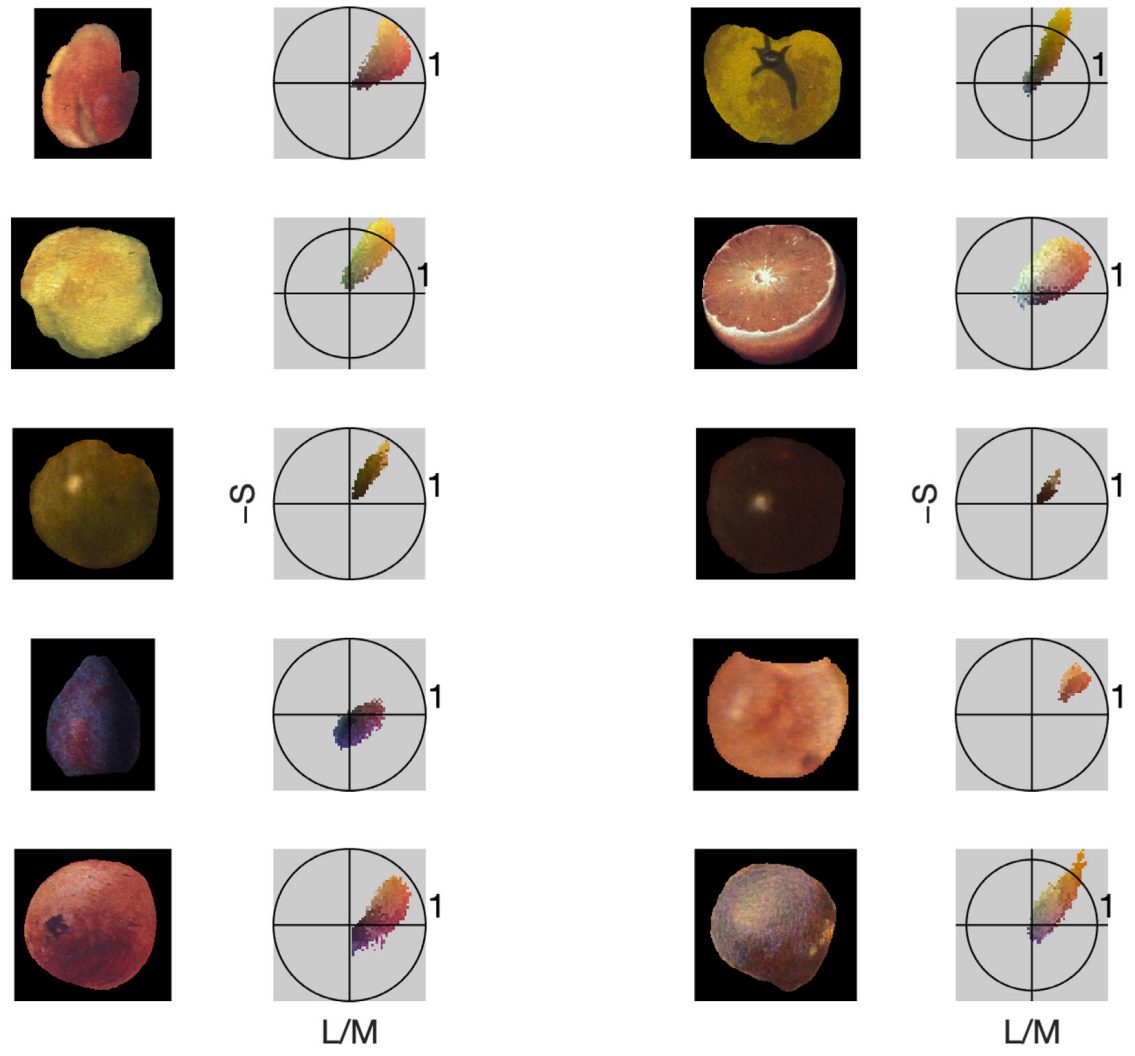
Joint distribution of image pixels of cut-outs of ten fruits chosen randomly from the paintings. The circle denotes unit contrast in the L/M vs. S plane. The results show that the pixels are disproportionately located in quadrant 1, supporting the contention that warm-colored pixels are a proxy for objects.

To investigate the memory color effect in paintings we focused on the relative distribution of pixels in the quadrant that contains fruit-colored pixels (quadrant 1, demarcated with the red arc, containing red, orange, and yellow colors) (Figures 3A and 3B) versus the three other quadrants (the black arc). The bottom of Figure 3 shows the relative number of pixels as a function of chromatic contrast. We confirm that fruit colors predominantly lie in the first quadrant by determining the joint distribution of image pixels of ten fruits chosen randomly from the paintings: The joint distributions show a strong bias for quadrant 1, the warm red–orange–yellow colors (Figure 4).

The number of relatively saturated pixels is greater in quadrant 1 than the other quadrants, for both paintings (Figure 3D) and photographs (Figure 3E; the red line extends further to the right of the black line and sits above it). This result shows that, in both paintings and photographs, the fruit colors are relatively more saturated. But the results also show that the relative difference of the number of saturated pixels in quadrant 1 versus the other quadrants is greater for the paintings than the photographs (the red line is further from the black line for the paintings than the photographs) (Figures 3D and 3E). This result is quantified in Figure 3F, in which the pink trace depicts for the paintings the relative difference in the number of pixels at each chromatic-contrast level for pixels in quadrant 1 versus the other quadrants, and the blue trace shows the same quantity for the photographs. A comparison of Figures 3A and 3B also shows a relative increase in purple (quadrant IV) in the paintings compared with the photographs, which may be explained by the increase of violet pigment in the late 19th century (Reutersvärd, 1950).

**Figure 5.**
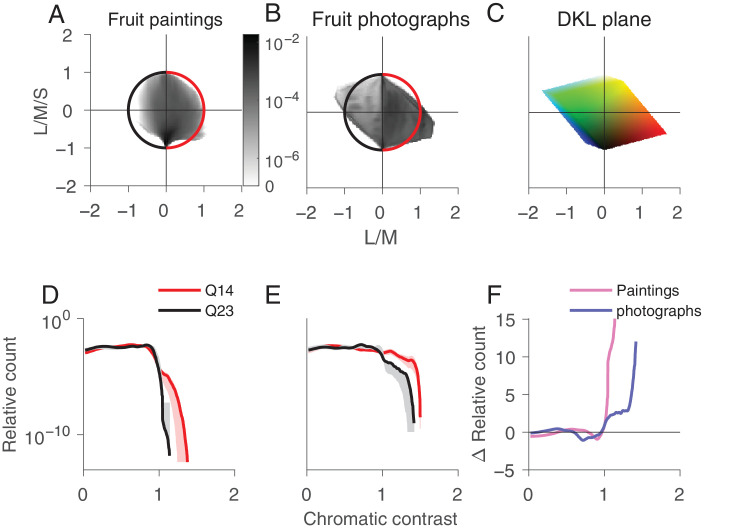
Analysis of color distribution in the L/M–L/M/S plane for paintings vs. photographs of fruits. Format is the same as in Figure 3. The dark spots in the histogram of the fruit photographs result from the cyan box shown as the second image in Figure 1B.

Prior work has shown that the parts of scenes that are more likely to be labeled as objects are warm colored rather than cool colored, for both natural objects and artificial objects (Rosenthal et al., 2018). Accordingly, to test the hypothesis that objects are relatively more saturated than backgrounds in still life paintings, we computed the joint histograms for the L/M vs L/M/S plane. We found that the warm +L/M colors are relatively more saturated than the cool colors (Figure 5).

**Figure 6.**
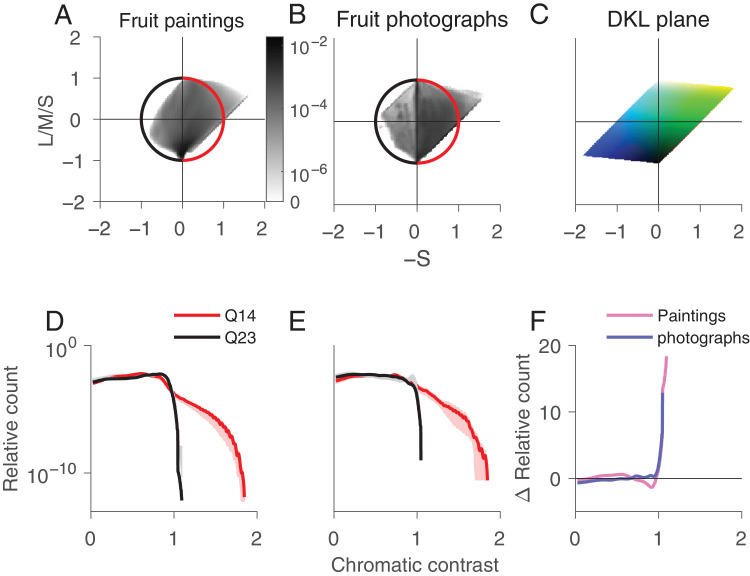
The frequency of “blue” pixels is almost identical in still-life paintings of fruit and photographs of fruit. Analysis of color distribution in the S–L/M/S plane for paintings vs. photographs of fruits. Format is the same as for Figure 3. The “blue” pixels plot in Quadrant 3.

We note that the results are also consistent with the conclusion that cool colors, typically associated with backgrounds, are relatively less saturated in the paintings. Blue is a commonly preferred color (Eysenck, 1941; McManus, Jones, & Cottrell, 1981; Palmer & Schloss, 2010), so if the effect we found for “warm” colors simply reflects color preference, it should also show up for the “blue” pixels. To test this hypothesis, we computed the joint histograms for the S plane versus the L/M/S plane and evaluated the relative chromatic contrast of pixels in the “blue” quadrant (Figure 6). As shown in Figure 6, the relative chromatic contrast of blue pixels versus other colored pixels is no greater for paintings than photographs. We conclude that the effect we found is specific for colors in the first quadrant and cannot be explained by color preference.

**Figure 7.**
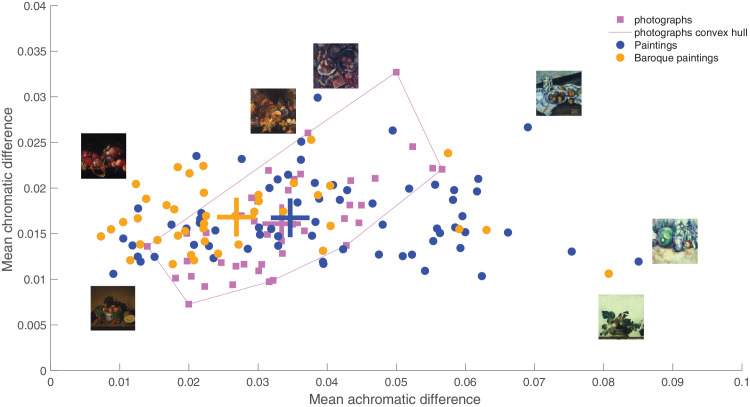
Analysis of the chromatic and achromatic difference in paintings (blue and orange dots) and photographs (pink squares).

Finally, we analyzed the chromatic and achromatic differences across the images, inspired by prior work that hypothesizes that visual discomfort is related to the local differences in luminance and chromaticity across an image (Penacchio et al., 2021). Penacchio et al. found support for this hypothesis in paintings and photographs of natural scenes, with a notable exception of fruit. The data set we analyzed is enriched for representations of fruit, providing an opportunity to further explore this potential exception and the generalizability of the hypothesis. Figure 7 shows for the still life paintings and the photographs the differences in CIE chromaticity (L, u′, and v′; as done by Penacchio et al.). The mean achromatic vs chromatic differences for photographs and paintings are indicated by the large crosses. The achromatic and chromatic differences are highly correlated for photographs, *r* = 0.68; *p* < 1e-6, but not for paintings, *r* = 0.007; *p* = 0.48, suggesting that a cognitive act of the artists is engaged to decouple color contrast from luminance contrast. The results also suggest that paintings made more recently (more contemporary vs. baroque) have higher achromatic contrast (the blue dots to the right of the convex hull for the photographs). In addition, it also seems that paintings of a single painter sometimes cluster (like for Jan Davidsz. De Heem, the yellow dots around [0.01, 0.015]), suggesting that this parameter may reflect an artistic fingerprint. Interestingly, the outlier of the Baroque paintings is Caravaggio's *Basket of Fruit*, which is the only painting that Caravaggio painted with a light background.

## Discussion

In the 17th century, the French Academy instituted a ranking of painting themes (Félibien, 1676). Still life painting was considered the least important, after history painting, portraiture, scenes of everyday life, and landscapes. Its rank reflected the view that still life painting is not the best vehicle to express the most important ideas of human experience. Instead, still life painting was undertaken as an exercise to work out how to paint—not just to see, but to observe. As such, still life paintings comprise a wealth of data that may be especially valuable for understanding visual perception (Toscani, Wolf, Gegenfurtner, & Braun, 2023). Herein, we analyzed the relative chromatic contrast of fruit versus background in still life paintings and photographs, using warm-colored pixels as a proxy of fruit and cool-colored pixels as a proxy for backgrounds (Rosenthal et al., 2018). The results show that the warm-colored regions of both paintings and photographs have relatively higher chromatic contrast than the cool-colored regions, and this relative difference is greater for the paintings, supporting the hypothesis that artists, on average, use relatively higher chromatic contrast to represent fruit.

Our project is motivated by questions regarding the relationship of color and shape and how this information is computed by the brain to form object concepts. On the one hand, several observations imply that the brain has separate circuits for computing color and shape. Human toddlers show a different time course for learning about colors and shapes (Wilcox, 1999; Prevor & Diamond, 2005), and adults can readily make independent reports about the color and the shape of objects. On the other hand, color appearance depends upon the spatial structure of the retinal image—the shape and arrangement of colors somewhat determine their appearance (Chevreul, 1854; Conway, 2001; Land, 1977). The semantic meaning of a color is similarly dependent on the shape context. Red can mean anger or love, depending on the shape it is attached to. One hypothesis is that the shape–contingency of color appearance is accomplished through experience, an idea that has been tested by evaluating cognitive penetration (Pylyshyn, 1999). According to cognitive penetration, knowledge of the colors of color-associated shapes impacts the perception of the colors of the shapes even when the shapes are rendered objectively colorless, for example, as a gray photograph.

Prior work has shown that achromatic renderings of familiar fruits tend to appear slightly colored with the typical color of the fruits (Hansen et al., 2006; Olkkonen et al., 2008). Participants tasked with adjusting digitally displayed colored images of fruit to appear achromatic tend to render the objects objectively colored, with a color that is roughly complementary to the typical color of the object. The explanation has been that this additional color is required to cancel the implicit memory color that unavoidably tints an achromatic rendering of a familiarly colored object. We reasoned that artists making paintings of still life paintings must hold in memory, even if briefly, the color of the objects they are looking at while painting, and this memory engagement, including the knowledge of the colors of the objects they are painting, impacts how they render the things they paint.

The idea that cognition affects perception has been challenged (Firestone & Scholl, 2016); the star piece of evidence in the challenge is the so-called El Greco fallacy (Firestone, 2013). As Firestone recounts, an ophthalmologist in 1913 suggested that distortions akin to those characteristic elongations in El Greco's paintings could be achieved by an astigmatic lens. The doctor recognized that an astigmatism in El Greco was an unlikely explanation for El Greco's painting style, but that did not stop the idea from taking root. The fallacy of the explanation, recognized by the doctor himself, is that if El Greco saw the world through an astigmatism, then he would not need to paint scenes as elongated—the scenes would already appear that way; adding real elongation would make them appear even more distorted. (The distortions in El Greco's work can easily be explained if the canvas was not perpendicular to the direction of gaze, as often happens when working on large canvases.) One might argue that the memory color effect advances a similar fallacy, because a perceptual or cognitive distortion that makes real objects appear in exaggerated saturation would also make the painted depictions appear exaggerated in saturation. However, a dismissal of the impact of perceptual or cognitive factors on art production comes too quickly.

Consider a situation where an image is viewed through a defocusing lens that makes the image blurry. A person viewing an in-focus picture through the blurry lens will see a blurry picture. Without access to the information lost by the blur of the lens, the person will make a reproduction that is different from the original and likely blurrier. This is a violation of the El Greco fallacy explanation. Or consider a more cognitive example, namely, the use of lines by artists. By the El Greco fallacy account, artists should not use lines because high-contrast linear elements do not typically structure natural scenes—if “seeing in lines” is a kind of lens through which we view the world, then we should not need to reproduce the world with lines because the world already “appears” as if it comprises lines. Of course, the standard account of line use by artists is that the lines activate the visual system in a way that matches how the real world does, and using lines is easier than accurately reproducing the retinal image: from a cognitive perspective, the lines represent the object concept, and this concept is what the artist is depicting. The failure of the El Greco fallacy is underscored by other observations, too. For example, it has recently been shown that biased perception of saturation can differ for each exemplar in a display showing multiples of the same object (Dubova & Goldstone, 2022), presumably because perception depends on an interaction of sensory signals with cognitive factors, such as attention and memory.

We reasoned that, even when artists are engaged in veridically representing a scene (whatever veridical might mean), they are inevitably influenced by the concepts of the things they are depicting. If true, we predicted that artists should unwittingly depict the fruit as more saturated than it is, in the same way that they tend to overemphasize lines and edges, because these attributes are, like colors, distinctive features of object concepts, used by the visual system to encode and remember objects. The results are consistent with this interpretation, suggesting that apparent chromatic contrast reflects processes of perception, cognition, attention, and memory. The direction of the effect we observed, in which the warm-colored pixels in the paintings (a proxy for the fruit) are relatively more saturated than the backgrounds (as compared with the photographs) is consistent with work showing that cognitive factors impact perception of the object to be matched, not the match (Dubova & Goldstone, 2022). In painting a still life, the object to be matched is the real fruit, and the match is the painting of it. The prediction from the work by Dubova and Goldstone (2022) is that a piece of real fruit having the same objective saturation as a depiction of it in a painting should seem to be more saturated than the painted version. So, the artists must exaggerate the saturation in the paintings to create a match, just as we observe.

Although we favor a cognitive-penetration interpretation, there are alternative possibilities. For example, the artists may have chosen relatively higher saturation for the warm colors over the cool colors for aesthetic reasons, or perhaps because of the impact of adaptation. The visual system adapts to chromatic conditions (Webster, 2015), so extended viewing of the image they are creating may make the colors appear less saturated. An adaptation account would, on first blush, predict exaggerated saturation for all colors in a still life, not just warm colors. However, it is possible that an explanation invoking an interaction of adaptation and cognitive factors could generate a disproportionate bias for warm over cool colors.

Regardless of the explanation, the present work underscores prior conclusions that perceived saturation cannot be mapped directly onto a physical attribute, but is instead the result of an interaction of perceptual and cognitive factors that reflect mechanisms of perception along with memory and behavioral demands. For example, memory colors are not obligatorily observed for achromatic reproductions of familiar objects. Achromatic representations of objects (photographs or digital displays), and the experience of objects under monochromatic light (which disables retinal color-encoding mechanisms) (Hasantash, Lafer-Sousa, Afraz, & Conway, 2019), are not perceived as colored in their usual colors—the achromatic photograph of a banana may, by some accounts, appear tinged with yellow but it does not appear as yellow as a real ripe banana. There are good reasons to doubt that objects should always be seen in their typical colors. If they were, color would be rendered useless as a cue to the state of an object. The memory color effect, however, is more subtle and may help to highlight objects that are important for us, by slightly modulating the feed-forward chromatic signal by prior knowledge.

Memory colors also seem to differ as a function of the color of the objects. Witzel et al. (2011) tested artificial objects such as an image of an orange comic mouse famous in Germany, a Smurf, and a Coke can. Memory colors were significant for blue and yellow objects, weak for orange and brown objects, and paradoxical for red objects. The relatively stronger memory color effects for blue and yellow objects may be explained by the requirement of some uncertainty in the sensory stimulus, such as the chromaticity of the lighting (Witzel, Olkkonen, & Gegenfurtner, 2016). The chromaticity of natural lights varies from blue to yellow, so there is inherent uncertainty about the extent to which a blue or yellow chromatic signal should be applied to the object or the lighting. A Bayesian framework, constrained by efficient encoding (Ganguli & Simoncelli, 2014; Wei & Stocker, 2015; Hasantash et al., 2019), has also been used to explain the paradoxical memory colors of reddish objects, including faces (Hasantash et al., 2019). One intriguing idea is whether the extreme familiarity of the color of faces establishes a prior about skin color that can be used to infer the illuminant (Crichton, Pichat, Mackiewicz, Tian, & Hurlbert, 2012; Lafer-Sousa & Conway, 2017; Chauhan, Xiao, & Wuerger, 2019). Taken together, both empirical and theoretical results lend weight to the idea that memory colors reflect important processes of conscious visual experience.

The slides of the paintings, although not calibrated, were taken by art historians to accurately represent the color in the paintings. Most of the paintings we analyzed were made during the baroque period (1593–1728) and later (1821–1943). Future work, with a larger data set, would allow an assessment of the validity of the results for specific time periods and art movements. Because our hypothesis regarding the exaggerated chromatic contrast of fruit colors versus non-fruit colors (or warm regions compared with cool regions) involves implicit processes, we expect that the effect will be somewhat independent of the period in which the work is made, just as line drawings are a potent way of capturing objects regardless of art period. But obviously artistic freedom has spawned a multitude of approaches, with some, such as expressionism, striving sometimes to decouple object color from optical representation. Future work using calibrated images would also help test the possibility of a systematic bias in how colored slides for art historical purposes are taken. We are alerted to the possibility of such a bias by the history of film: early film technology favored the contrast distribution of Caucasian faces (Dyer, 1997).

Finally, we analyzed chromatic and luminance differences in both the still life paintings and the photographs. We were motivated in this analysis to show how future analysis of art works could provide insight into mechanisms of perception and cognition, inspired by work showing that chromatic differences and luminance differences are correlated in natural scenes; increases in these differences are associated with increases in visual discomfort (Penacchio et al., 2021). Penacchio et al. showed that high chromatic differences are relatively rare in natural scenes, with a noted exception being images of fruit. Our analysis, which focused on fruit, shows that chromatic differences and luminance differences were only correlated in the photographs, not the still life paintings: the artwork shows a large range of luminance differences and a relatively invariant, and smaller range, of chromatic differences. This result suggests that artists can decouple luminance and chromatic differences that are otherwise bound in natural images, which could imply that luminance and color are constructed concepts—outputs of the brain, not inputs to it, as often assumed. The artistic practice is consistent with the idea that visual discomfort is triggered more strongly by chromatic differences than luminance differences, an idea that warrants further study.

## Appendix

**Table 1.** List of paintings

1	aelst_stlbasketmarble1650.tif	:	Aelst	(1650)	Baroque
2	aelst_stlfruit.tif	:	Aelst	(1652)	Baroque
3	aelst_stlfruit1667.tif	:	Aelst	(1667)	Baroque
4	andrieu_stlfruitflow.tif	:	Andrieu	(NaN)	Baroque
5	ast_fruitbask1632.tif	:	Ast	(1632)	Baroque
6	ast_shellsfruit1937.tif	:	Ast	(1637)	Baroque
7	ast_stlfruitflowbask1623.tif	:	Ast	(1623)	Baroque
8	baschenis_stlfruit.tif	:	Baschenis	(NaN)	Baroque
9	beckmann_pearsorchids1946.tif	:	Beckmann	(1946)	Expressionism
10	beert_cherries.tif	:	Beert	(NaN)	Baroque
11	beert_stlstrawberries.tif	:	Beert	(NaN)	Baroque
12	benton_stlfruitveg1914.tif	:	Benton	(1914)	Expressionism
13	bergoijs_banqfruitpew.tif	:	Bergoijs	(NaN)	Baroque
14	bonnard_intstlfruit1923.tif	:	Bonnard	(1923)	Expressionism
15	bonnard_stlbasket1936.tif	:	Bonnard	(1936)	Expressionism
16	bonnard_stlbowl1933.tif	:	Bonnard	(1933)	Expressionism
17	bonnard_stleve1926.tif	:	Bonnard	(1926)	Post-impressionism
18	bonnard_stlfruit1936.tif	:	Bonnard	(1936)	Post-impressionism
19	braque_stlbowlfruit1926.tif	:	Braque	(1926)	Expressionism
20	braque_stlbrownpitch1927.tif	:	Braque	(1927)	Expressionism
21	braque_stlfruitglassbot1924.tif	:	Braque	(1924)	Expressionism
22	braque_stlfruitstringed1938.tif	:	Braque	(1938)	Expressionism
23	carvag_baskfruit1596.tif	:	Caravaggio	(1599)	Baroque
24	carvag_boywithbask1594.tif	:	Caravaggio	(1593)	Baroque
25	cezanne_applessugar1906.tif	:	Cezanne	(1906)	Post-impressionism
26	cezanne_cherriespeaches1887.tif	:	Cezanne	(1887)	Post-impressionism
27	cezanne_compotier1894.tif	:	Cezanne	(1894)	Post-impressionism
28	cezanne_fruitappbread1880.tif	:	Cezanne	(1880)	Post-impressionism
29	cezanne_fruitgingbask1890.tif	:	Cezanne	(1890)	Post-impressionism
30	cezanne_gingerjar1893.tif	:	Cezanne	(1893)	Post-impressionism
31	cezanne_greenmelon1906.tif	:	Cezanne	(1906)	Post-impressionism
32	cezanne_jugfruit1894.tif	:	Cezanne	(1894)	Post-impressionism
33	cezanne_milkpitcherfru1900.tif	:	Cezanne	(1900)	Post-impressionism
34	cezanne_pom1906.tif	:	Cezanne	(1906)	Post-impressionism
35	cezanne_potsbottcup1871.tif	:	Cezanne	(1871)	Post-impressionism
36	cezanne_stlapples1898.tif	:	Cezanne	(1898)	Post-impressionism
37	cezanne_stlappleschair1906.tif	:	Cezanne	(1906)	Post-impressionism
38	cezanne_stlbottlespeach1894.tif	:	Cezanne	(1894)	Post-impressionism
39	cezanne_stlcommode1888.tif	:	Cezanne	(1888)	Post-impressionism
40	cezanne_stlflowfruit1890.tif	:	Cezanne	(1890)	Post-impressionism
41	cezanne_stlfruitflow1890.tif	:	Cezanne	(1890)	Post-impressionism
42	cezanne_stlfruitwine1879.tif	:	Cezanne	(1879)	Post-impressionism
43	cezanne_stlgingerjar1894.tif	:	Cezanne	(1894)	Post-impressionism
44	cezanne_stlpeachpear1887.tif	:	Cezanne	(1887)	Post-impressionism
45	cezanne_stlpears1893.tif	:	Cezanne	(1893)	Post-impressionism
46	cezanne_stlpitchsugar1885.tif	:	Cezanne	(1885)	Post-impressionism
47	cezanne_sugarpotbluecup1866.tif	:	Cezanne	(1866)	Post-impressionism
48	chardin_buffet1738.tif	:	Chardin	(1728)	Baroque

**Table 1.** Continued

49	claesz_breakfasttable1630.tif	:	Claesz	(1630)	Baroque
50	claesz_fruitstillgilded1661.tif	:	Claesz	(1661)	Baroque
51	claesz_stlberekemeyer11621.tif	:	Claesz	(1621)	Baroque
52	claesz_stlturkey1627.tif	:	Claesz	(1627)	Baroque
53	dumont_stldish1912.tif	:	Dumont	(1912)	Expressionism
54	duncanson_stlfruit1848.tif	:	Duncanson	(1848)	Realism
55	exter_cherries1914.tif	:	Exter	(1914)	Cubism
56	fyt_stlfruitfowl1662.tif	:	Fyt	(1662)	Baroque
57	gaugin_teapot1896.tif	:	Gaugin	(1896)	Expressionism
58	glackens_stlrosesfruit1924.tif	:	Glackens	(1924)	Post-impressionism
59	gogh_stlpears1889.tif	:	Van Gogh	(1889)	Post-impressionism
60	heem_abundantlife1655.tif	:	Heem	(1655)	Baroque
61	heem_champipe1642.tif	:	Heem	(1642)	Baroque
62	heem_fruitflowgarland1651.tif	:	Heem	(1651)	Baroque
63	heem_garland1653.tif	:	Heem	(1653)	Baroque
64	heem_shellfruithague.tif	:	Heem	(NaN)	Baroque
65	heem_stl1650.tif	:	Heem	(1650)	Baroque
66	heem_stlbirdsnest.tif	:	Heem	(NaN)	Baroque
67	heem_stlfruitflow.tif	:	Heem	(NaN)	Baroque
68	heem_stlfruitjapanbowl.tif	:	Heem	(NaN)	Baroque
69	heem_stlifetotal.tif	:	Heem	(NaN)	Baroque
70	heem_stlvinefruit.tif	:	Heem	(NaN)	Baroque
71	hunt_stlfruit1943.tif	:	Hunt	(1943)	Modern
72	johnson_stlbook1931.tif	:	Johnson	(1931)	Expressionism
73	kahlo_fruitoflife1953.tif	:	Kahlo	(1953)	Expressionism
74	kahlo_livinglife1951.tif	:	Kahlo	(1951)	Expressionism
75	kahlo_stl1952.tif	:	Kahlo	(1952)	Expressionism
76	kahlo_stlcereus1938.tif	:	Kahlo	(1938)	Expressionism
77	kahlo_stllonglivelife.tif	:	Kahlo	(NaN)	Expressionism
78	kahlo_stlparrotflag1951.tif	:	Kahlo	(1951)	Expressionism
79	ladell_stlfruitbutter.tif	:	Ladell	(NaN)	Baroque
80	ladell_stlfruitivorycask.tif	:	Ladell	(NaN)	Baroque
81	ladell_stlfruitledge.tif	:	Ladell	(NaN)	Baroque
82	matisse_peaches1940.tif	:	Matisse	(1940)	Expressionism
83	mignon_fruitfishnest1670s.tif	:	Mignon	(1670)	Baroque
84	mignon_stllife.tif	:	Mignon	(NaN)	Baroque
85	moillon_stlbaskasp1630.tif	:	Moillon	(1630)	Baroque
86	monet_spanishmelon1880.tif	:	Monet	(1880)	Impressionism
87	monet_stlflowerfruit1869.tif	:	Monet	(1869)	Impressionism
88	oudry_stlfruitscelery1725.tif	:	Oudry	(1725)	Baroque
89	peale_stlbowl1825.tif	:	Peale	(1825)	Renaissance
90	peale_stlcake1822.tif	:	Peale	(1822)	Renaissance
91	peale_stlfruit1821.tif	:	Peale	(1821)	Renaissance
92	pealej_stlfruitbowl1825.tif	:	Peale	(1825)	Renaissance
93	pealem_watermelon1828.tif	:	Peale	(1828)	Renaissance
94	pealer_stlfruit.tif	:	Peale	(NaN)	Renaissance
95	pealer_stlorange.tif	:	Peale	(NaN)	Renaissance
96	peploe_stlfruit1918.tif	:	Peploe	(1918)	Expressionism
97	ramirez_stlcardoon.tif	:	Ramirez	(NaN)	Renaissance
98	ramirezx_stlgrapesmelon.tif	:	Ramirez	(NaN)	Renaissance
99	ring_stlfruitoysters1934.tif	:	Ring	(1934)	Renaissance
100	roesen_naturesbeauty1855.tif	:	Roesen	(1855)	Renaissance

**Table 1.** Continued

101	roesen_stillife.tif	:	Roesen	(NaN)	Renaissance
102	roesen_stllifePAFA.tif	:	Roesen	(NaN)	Renaissance
103	roesen_stlwfruit1850.tif	:	Roesen	(1859)	Renaissance
104	ruysch_naturamorta.tif	:	Ruysch	(NaN)	Baroque
105	ruysch_stlfruitani1716.tif	:	Ruysch	(1716)	Baroque
106	soreau_stlfruit1638.tif	:	Soreau	(1638)	Baroque
107	velde_stlbeerdetails1647.tif	:	Velde	(1647)	Baroque
108	waldmuller_stlrosefruit1827.tif	:	Waldmuller	(1827)	Renaissance
